# A retrospective analysis of MS/MS screening for IEM in high-risk areas

**DOI:** 10.1186/s12920-023-01483-1

**Published:** 2023-03-16

**Authors:** Xiao He, Juan Kuang, Jiahong Lai, Jingxiong Huang, Yijin Wang, Guofeng Lan, Yingjun Xie, Xuekai Shi

**Affiliations:** 1 Department of Pediatrics, The Second Nanning People’s Hospital, Nanning, 530031 Guangxi China; 2 Department of Clinical Medicine, The Third Clinical School of Guangzhou Medical University, Guangzhou, 511436 Guangdong China; 3 Department of Obstetrics and Gynecology, Guangdong Provincial Key Laboratory of Major Obstetric Diseases, The Third Affiliated Hospital of Guangzhou Medical University, Guangzhou, 510150 Guangdong China; 4 Guangdong Provincial Key Laboratory of Major Obstetric Diseases, The Third Affiliated Hospital of Guangzhou Medical University, Guangzhou, 510150 Guangdong China

**Keywords:** Inborn errors of metabolism, Newborn screening, Tandem mass spectrometry, Genetic testing, Sequencing

## Abstract

Inborn errors of metabolism (IEM) can lead to severe motor and neurological developmental disorders and even disability and death in children due to untimely treatment. In this study, we used tandem mass spectrometry (MS/MS) for primary screening and recall of those with positive primary screening for rescreening. Further diagnosis was based on biochemical tests, imaging and clinical presentation as well as accurate genetic testing using multi-gene panel with high-throughput sequencing of 130 IEM-related genes. The screening population was 16,207 newborns born between July 1, 2019, and December 31, 2021. Based on the results, 8 newborns were diagnosed with IEM, constituting a detection rate of 1:2,026. Phenylketonuria was the most common form of IEM. In addition, seven genes associated with IEM were detected in these eight patients. All eight patients received standardized treatment starting in the neonatal period, and the follow-up results showed good growth and development. Therefore, our study suggests that MS/MS rescreening for IEM pathogenic variants in high-risk areas, combined with a sequencing validation strategy, can be highly effective in the early detection of affected children. This strategy, combined with early intervention, can be effective in preventing neonatal morbidity and improving population quality.

## Introduction

Inborn errors of metabolism (IEM) are a group of disorders caused by genetic pathogenic variants that lead to abnormalities in the function of enzymes, receptors, transport proteins, or membranes, which in turn lead to metabolic disorders in the body, resulting in pathological and clinical symptoms [1]. According to previous studies, the global birth rate of IEM is 50.9:100,000 live births [2]. IEM may present with nonspecific symptoms, such as vomiting, seizures, and respiratory distress, that overlap with many other diseases, such as sepsis, cerebral haemorrhage, and encephalitis [3]. This overlap makes early and immediate diagnosis of IEM difficult in some neonates, causing it to often be misdiagnosed as other diseases.

The types of diseases newborns are screened for vary by race, country and region and are also related to social, scientific and technological development, economic level and the degree of risk of the disease in each country. In China, screening for IEM focuses on diseases such as phenylketonuria (PKU) and congenital hypothyroidism (CH), while other regions screen for glucose-6-phosphate dehydrogenase (G6PD) deficiency, depending on the disease. Moreover, tandem mass spectrometry has been introduced for newborn screening for other rare genetic metabolic diseases, such as disorders of amino acid, organic acid and fatty acid metabolism. With the development of experimental diagnostic techniques, most screening laboratories in China have adopted fluorescence analysis (full quantitative) for PKU screening, and very few laboratories still use the traditional Guthrie bacterial inhibition method (semiquantitative) and high-performance liquid chromatography for PKU screening. Enzyme-linked immunoassay and enzyme immunofluorescence methods are available for CH screening. In the last decade, tandem mass spectrometry has been used in developed countries to screen for approximately 25 inherited metabolic defects, including disorders of amino acid, organic acid and fatty acid metabolism, greatly improving screening efficiency.

Currently, the main method of clinical screening for neonatal IEM is tandem mass spectrometry (MS/MS). Dozens of IEMs can be screened by analysing blood samples for concentrations of various amino acids, succinylacetone, free carnitine and acylcarnitine, such as some lysosomal storage disorders, ADA and PNP SCIDs, X-adrenoleucodistrophy (X-ALD), Wilson disease, guanidinoacetate methyltransferase deficiency (GAMT), and Duchenne muscular dystrophy [4].MS/MS has the advantage of rapid detection with high sensitivity, high specificity, high throughput (the ability to screen for 30~40 genetic metabolic diseases at once) and low sample volume (a few tens of microlitres of peripheral blood on a filter paper sheet, dried and sent for testing). At the same time, the price is relatively modest, and the test cycle time is short, which makes it suitable for promotion and popularization. However, the diagnostic reference index of IEMs varies by laboratory. In addition, whether the collection of dried blood spot samples is standardized can affect the results. Several studies have shown that the levels of most amino acids and acylcarnitine decrease over time at ambient temperature, which can lead to false-negative results [3]. In addition, due to the sensitivity of existing instruments, the quantitative analysis of some analytes requires advanced derivatization, which increases the analysis time and cost of use and is not conducive to large-scale clinical analytical applications. Additionally, substandard experimental instrumentation, laboratory personnel not following strict laboratory standard operating procedures, irregular collection, improper storage, damp filter paper, untimely delivery, and problems with the assay itself may all contribute to the false-positive/false-negative rate. Therefore, MS/MS combined with next-generation sequencing can compensate for the shortcomings of current NBS methods and reduce the false-positive/false-negative rate to a great extent, making the results more reliable [5].

In this study, 16,207 newborns were screened for neonatal IEM by MS/MS, and the positive cases were validated by next-generation sequencing technology, which reduced the false-positive. This detection strategy not only helps to identify new variant loci but also enables early detection of affected children, which combined with early intervention can effectively prevent neonatal morbidity.

## Materials and methods

**Fig. 1:**
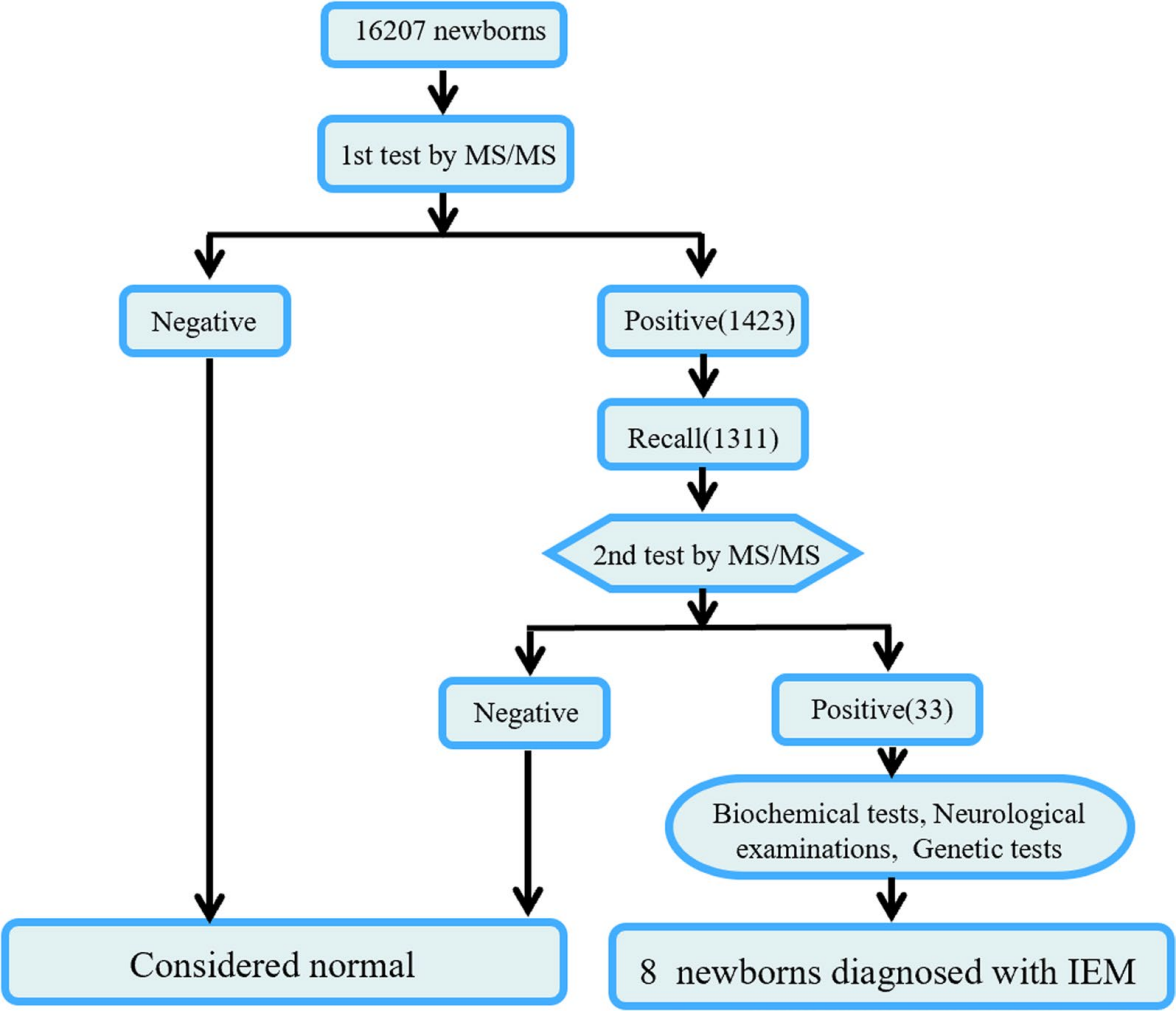
Flow chart of IEM screening and the diagnosis process

A total of 16,207 newborns borned in Nanning, Guangxi Province, China underwent newborn IEM screening by tandem mass spectrometry from July 1, 2019 to December 31, 2021. Newborns were all newborns who had completed 72 h after birth and had been fed adequately for at least 8 times. Other inclusion criteria were complete medical history. Exclusion criteria were: (1) Newborns undergoing emergency surgery; external blood transfusion. (2) Recent antibiotic or steroid use. (3) Maternal genetic metabolic disease, liver disease or HELLP (hemolysis, elevated liver enzymes, and low platelet count) syndrome. Data on the basic clinical information of the participants includes immediate post-vital assessment, Apgar score, examination for severe malformations and birth injuries, physical development assessment, gestational age assessment. Newborn parents signed an informed consent form. Data on IEM screening and diagnostic procedures were collected for the studied neonates (Fig. 1).

### Laboratory methodology

#### Collection and requirements of dried blood spots (DBS)

Using the newborn disease screening blood sample collection card, heel blood (filter paper dried blood spot specimen) was collected from newborns who were exclusively breastfed or bottle-fed with expressed breast milk more than 8 times within 72 h after birth in accordance with the requirements of the Technical Specification for Blood Spot Collection for Newborn Disease Screening (WS 376.3–2013). The requirements for filter paper DBS were as follows: blood spot diameter ≥ 8 mm, number of blood spots of 3~4, and uniform penetration on both sides. The DBS samples on filter paper were dried horizontally, sealed in sealed bags, and sent to the neonatal disease screening unit of the hospital's Laboratory Department for tandem mass spectrometry testing.

The Gesell Developmental Schedule was assessed for the child’s intellectual, motor and language development [6], and based on a series of longitudinal studies of the normal course of behavioural development in infants and preschool children. The schedules cover the major areas of behavioural development, namely motor, adaptive, language, personal and social development. It involves a standardised procedure for observing and assessing the various elements of the child's behaviour. It includes tests as well as observations of the child. These schedules are applicable to children from 4 months to 6 years of age [3].

#### Tandem mass spectrometry screening method

Tandem mass spectrometry for IEM was conducted as follows: Researchers in the tandem mass spectrometry laboratory strictly followed the operating instructions of the kit (nonderivative multiple amino acid, carnitine and succinylacetone assay kits) and the operating procedures of the tandem mass spectrometer (Waters TQD tandem mass spectrometry analysis system, Waters Corporation, USA). A 3-mm diameter blood sample was prepared from a neonatal dried blood spot sample using a punch clamp and reagents in the kit [7]. The concentrations of amino acids and carnitine (free carnitine and acylcarnitine) and the ratios between amino acids and carnitine (free carnitine and acylcarnitine) were analysed by tandem mass spectrometry. Newborns with abnormal amino acid or carnitine (free carnitine and acylcarnitine) indices were recalled for recollection of heel blood (filter paper dried blood spot specimens) for rescreening. If the results of both screens were MS/MS-positive, the newborn was judged to be suspected of IEM.

#### Quality control of tandem mass spectrometry screening IEM

The kit quality control products used for laboratory quality control in this study were classified as low and high levels. The QC frequency was tested with unknown samples in each experiment. QC rules were used in a Z score QC chart with 12 s as the warning limit and 13 s as the out-of-control limit. Standard deviations were calculated from the QC data of this experiment. The method to address the loss of control is to take various effective corrective measures according to the standard operating procedures of indoor quality control and correct them in time. After confirming that the control status is restored, the sampling test can be started. When 20 data points have not been accumulated to establish a quality control chart, the Grubbs method should be used immediately for internal quality control. Moreover, external quality assessment (EQA) is conducted regularly every year.

The experimental reports were reviewed by IEM experts with genetic consultant qualifications from the Second Nanning People's Hospital, China.

#### Diagnosis of suspected IEM

For MS/MS-positive newborns with suspected IEM, blood biochemistry, heavy tandem mass spectrometry and genetic analysis were used for diagnosis. The genetic analysis method was high-throughput panel sequencing: after obtaining informed consent from the guardians, 2 ml of peripheral blood from the suspected children and their parents was drawn and sent to Shenzhen United Medical Technology Co., Ltd. for high-throughput sequencing of 130 IEM-related genes (NextSeq500, Illumina). The target sequences were captured by high-multiplicity PCR amplification covering all exonic regions and adjacent intronic regions (± 50 bp) of the target genes. For children with suspected gene positivity, Sanger sequencing was used for locus validation and parental verification.

## Results

### The screening results, diagnosis, and follow-up of neonatal IEM

Of the 16,207 newborns, 1423 were positive upon primary screening (8.78%). Moreover, 772 newborns (54.25%) were male and 651 (45.75%) were female. In total, 378 (26.56%) were preterm (< 37 weeks of gestation), and 1045 (73.44%) were full-term (≥ 37 weeks of gestation). Amino acid abnormalities, such as decreased arginine and citrin and increased tyrosine (8.08%, 7.38% and 7.03% of positives, respectively), and carnitine abnormalities, such as decreased free carnitine and octenyl carnitine and increased free carnitine and alanine (16.59%, 12.86%, 12.30% and 7.73% of positives, respectively), were more common in those with positive primary screening (Table 1).

**Table 1:** Distribution of 1423 newborns with IEM positive in primary MS/MS screening

Indicators (reduction/elevation)	Normal reference range (μmol /L)	Abnormal range values (μmol /L)	Overall mean (μmol /L)	The number of cases	Percentage (%)
*Amino acids*					
Arginine reduction	2.00–50.00	0.50–1.00	9.52 ± 31.24	115	8.08
Citrulline reduction	7.90–37.00	3.34–7.64	12.70 ± 7.99	105	7.38
Tyrosine elevation	34.50–250.00	256.02–1177.78	104.94 ± 43.66	100	7.03
Glycine reduction	246.57–1283.00	100.81–202.23	436.43 ± 130.25	52	3.65
Methionine elevation	7.18–41.35	42.12–898.81	22.43 ± 12.40	49	3.44
Phenylalanine elevation	23.30–120.00	24.04–1299.87	57.43 ± 21.07	58	4.08
*Carnitines*					
Free carnitine reduction	10.28–54.24	0.76–5.14	22.56 ± 65.29	236	16.59
Octenylcarnitine reduction	0.05–0.43	0–0.03	0.07 ± 0.03	183	12.86
Free carnitine elevation	10.28–54.24	100.02–7178.07	22.56 ± 65.29	175	12.30
Propionyl carnitine elevation	0.43–3.80	7.60–16.73	1.83 ± 0.81	110	7.73
Succinyl acetone elevation	0–0.92	1.82–21.35	0.69 ± 0.31	49	3.44
Others				191	13.42

A total of 1311 newborns were recalled and rescreened because of their positive primary screening results, for a recall rate of 92.21%. In total, 33 cases were suspected to be IEM based on the results of rescreening by blood tandem mass spectrometry. In addition, 8 cases of IEM were diagnosed based on biochemical tests, neurological tests (using Magnetic Resonance Imaging (MRI)) and genetic tests, implying a prevalence of 1:2,026 among newborns in the study area and during this study period. These cases included 4 cases of amino acid metabolic disease (50.00%): 2 cases of PKU, 1 case of citrin deficiency and 1 case of tyrosinemia type I (HT-1); 2 cases of organic acid metabolic disease (25.00%):1 case each of methylmalonic acidemia (MMA) and glutaric aciduria type I (GA-I); 2 cases (25.00%) of fatty acid oxidation disorders: 1 case each of carnitine palmitoyltransferase II (CPT II) deficiency and primary carnitine deficiency (PCD) (Table 2).

**Table 2:** Screening and diagnosis of 16,207 newborns with IEM in this study

Disease*	Number of confirmed cases	Incidence rate	Composition ratio (%)	Interventions (for reference)	Symptoms#
*Amino acid metabolic disease*	4	1:4,052	50.00		
PKU	2	1:8,104	25.00	Low phenylpropyl amino acid formula milk was given in early treatment. No medication administered	Normal
Citrin deficiency	1	1:16,207	12.50	A lactose-free, rich medium-chain fatty acids dietetic treatment	Normal
HT-1	1	1:16,207	12.50	Special formula without Tyr and Phe, mixed with breast milk daily, and oral nitisinone and hepatoprotective therapy to maintain plasma Tyr below 400 μmol/L and Phe below 120 μmol /L	Normal
*Organic acid metabolic disease*	2	1:8,104	25.00		
MMA	1	1:16,207	12.50	Special formula that restricted natural protein and supplemented with special formulas that removed valine, isoleucine, methionine and threonine, as well as levocarnitine (50–100 mg/kg. bid) and oral vitamin B12 (10 mg/kg. qd)	Normal
GA-I	1	1:16,207	12.50	A high protein restricted diet, and treatment with oral vitamin B2 (5 mg/kg.tid) with L-carnitine (50~100 mg/kg. bid)	Head circumference slightly larger, cranial MRI showed normal and no neurological developmental deficits
*Fatty acid oxidation disorder*	2	1:8,104	25.00		
CPT II deficiency	1	1:16,207	12.50	L-carnitine (50~100 mg/kg. bid) and formula supplemented with medium- and long-chain fatty acids to maintain stable levels of free carnitine in the blood	Normal
PCD	1	1:16,207	12.50	Treated with oral L-carnitine (50~100 mg/kg. bid) and milk powder with medium- and long-chain fatty acids	Normal
Total	8	1:2,026	100.00		

*PKU Phenylketonuria; *HT-1* Hereditary tyrosinemia type I; *MMA* Methylmalonic acidaemia; *GA-I* Glutaric aciduria type I; *CPT II* Carnitine palmitoyltransferase II; *PCD* primary carnitine deficiency

^#^Follow-up until 2–3 years of age on the child's intellectual, motor and language development

Telephone callbacks to 112 families who were not recalled for rescreening (cut-off date December 31, 2021) showed that 22 parents of newborns refused to answer the telephone, and the guardians of the remaining newborns responded that the infants were currently healthy.

**Fig. 2:**
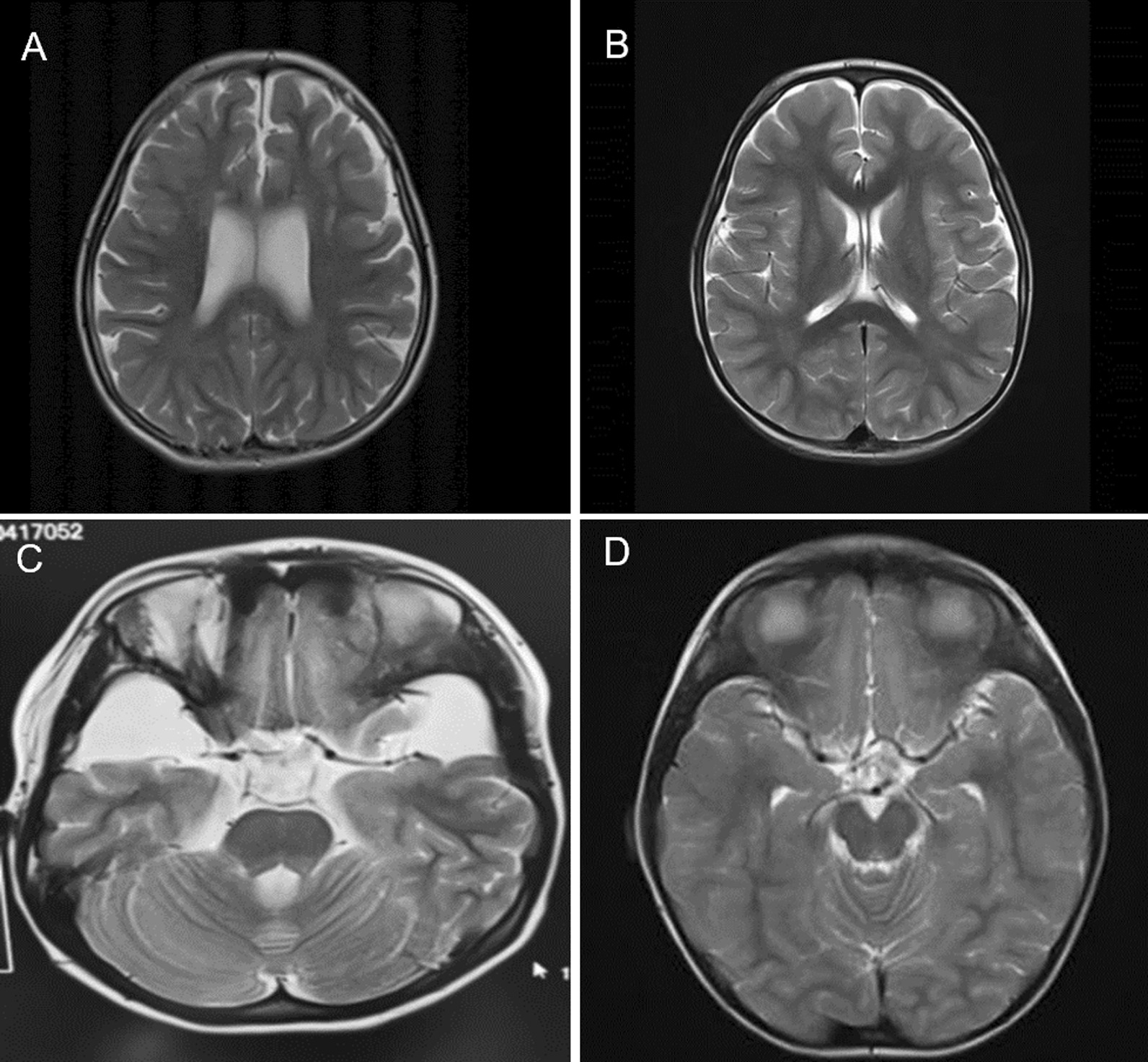
Characteristics of cranial MRI in a patient with methylmalonic acidemia (**A**, **B**) and a patient with glutaric acidemia (**C**, **D**). **A**: Iso-T1 and long T2 signals shown in the white matter of the frontal lobes, bilateral frontal white matter demyelination enhancement and mild dilatation of the lateral ventricles (at 3 months of age). **B**: Normal (at 2 years of age). **C**: The bilateral temporal subarachnoid space was significantly enlarged (at 3 months of age). **D**: Normal (at 2.5 years of age)

We followed 8 newborns diagnosed with IEM and assessed their intellectual, motor and language development using the Gesell Developmental Schedule. One patient with methylmalonic acidaemia showed abnormal signals in the bilateral pallidum and bilateral caudate nuclei on cranial MRI (Fig. 2A, B), and 1 patient with glutaric acidaemia showed iso-T1 and long T2 signals in the frontal white matter on early cranial MRI, suggesting increased bilateral frontal white matter demyelination (Fig. 2C, D). All patients were given appropriate treatment, including specialized milk powder and supplementation with L-carnitine (50~100 mg/kg. bid), vitamin B 2 (5 mg/kg. tid), vitamin B12 (10 mg/kg.qd), and nitroprusside. Currently, they are all growing well according to the 2-year follow-up data. At the age of 2 years, their cranial MRI was normal, and their intelligence was normal (Table 2).

### Genetic analysis of newborns with suspected IEM

Thirty-three newborns with suspected IEM were tested for genetic abnormalities, and 8 of them were diagnosed with IEM due to the detection of associated genetic pathogenic variants. Of these, CPT II deficiency, MMA, and GA-I were all parentally verified compound heterozygous pathogenic variants. The remaining five cases of IEM were homozygous pathogenic variants, consistent with the genetic characteristics of autosomal recessive disorders (Table 3).

**Table 3:** Variation information of 8 children with gene-positive IEM

Case	Confirmed disease*	Gene	Variation site	Homozygous/Heterozygous	Pathogenicity analysis^#^
1	CPT II deficiency	*CPT2*	c.989dupT (p. Ile332HisfsTer2)	Compound heterozygosity	Pathogenic
			c.1393G > A (p. Ala465Thr)		Unclear significance
2	MMA	*MUT*	c.729_730insTT (p. Asp244LeufsTer39)	Compound heterozygosity	Pathogenic
			c.424A > G (p.Thr142Ala)		Unclear significance
3	Citrin deficiency	*SLC25A13*	c.852_855delTATG (p. Met285ProfsTer2)	Homozygous variation	Pathogenic
4	HT-1	*FAH*	c.455G > A ( p.Trp152*)	Homozygous variation	Pathogenic
5	PKU	*PAH*	c.158G > A (p.Arg53His)	Homozygous variation	Pathogenic
6	PKU	*PAH*	c.728G > A (p.Arg243Gln)	Homozygous variation	Pathogenic
7	PCD	*SLC22A5*	c.1400C > G (p. Ger467Cys)	Homozygous variation	Pathogenic
8	GA-I	*GCDH*	c.1109 T > C (p. Leu370Pro)	Compound heterozygosity	Pathogenic
			c.395G > A (p. Arg132Gln)		Pathogenic

*CPT II Carnitine palmitoyltransferase II deficiency; *MMA* Methylmalonic acidaemia; *HT-1* Hereditary tyrosinemia type I; *PKU* Phenylketonuria; *PCD* primary carnitine deficiency; *GA-I* Glutaric aciduria type I

^#^According to ACMG standards and guidelines

## Discussion

Our study applied tandem mass spectrometry to screen for IEMs and found an overall prevalence of 1:2026 in Nanning, which is higher than the overall national level in China. As in China, the distribution of IEM varies by geographic location, with an estimated total IEM incidence of 17.14:100,000 births, or approximately 1:5834 [8]. Compared with the incidence of IEM detected by MS/MS in other regions of China, the incidence in Nanning was significantly higher than that in Liuzhou (1:3733) [9], Beijing (1:3666) [10], Suzhou (1:3163) [11], Changsha (1:4237) [12], and Hong Kong (1:4122) [13] and lower than that in Xinxiang (1:1617) [14] and Jining (1:1941) [15]. In addition, the incidence of IEM detected by MS/MS in the NBS study varied considerably between countries. Collectively, the estimated IEM incidence rates were 1:4000 in the United States [16], 1:8557 in Japan [17], 1:2800 in South Korea [18], 1:2400 in Germany [19], 1:1482 in Lebanon [20], and 1:2060 in Spain [21]. Notably, countries from the Middle East and Northern Africa, such as Lebanon, have a high incidence of IEM because of the demographic characteristics of high consanguinity (25~70%) and a high percentage of cousin marriages [22]. Most IEMs are predominantly recessive in inheritance. High consanguinity and consanguineous marriages increase the risk of IEM [23], which is also seen in Pakistan [24]. In addition, ethnicity, degree of economic development, disease spectrum and genetic background, screening methods and procedures, laboratory screening capacity, and number of diseases screened for NBS are significantly associated with differences in the incidence of IEM across countries [2, 25]. In our study, the Nanning area is home to the Zhuang minority and intermarriage has been common throughout history, and consanguineous marriages are high, which is the reason for the increased prevalence of IEM in Nanning compared to the rest of China.

We diagnosed 4 newborns with disorders of amino acid metabolism, representing 62.50% of all diagnosed cases and the most common type of IEM in this screening. These cases included two cases of PKU, one case of citrin deficiency and one case of HT-1. In this study, 2 children with PKU had blood Phe concentrations > 120 μmol/L (2 mg/dl) and both Tyr and Phe were above 2 times the normal reference value.Their pathogenic variants were mainly homozygous, characterized by c.1068C > A and c.728G > A. After early treatment with a low Phe formula and follow-up until 3 years of age, these children with PKU had good intellectual, motor and language development. Citrin deficiency is an autosomal recessive disorder caused by pathological variants in the *SLC25A13* gene. It can impair glycolysis and de novo lipogenesis in the liver leading to a state of energy deficit. The primary clinical manifestation in infancy is neonatal intrahepatic cholestasis caused by citrin deficiency (NICCD), which may present with growth impairment, persistent jaundice, abnormal liver function, and hepatomegaly. In our study, the child with citrin deficiency has a homozygous frameshift mutation in the *SLC25A13* gene: c.852_855delTATG (p.Met285ProfsTer2).This mutation may lead to the loss of gene function and belongs to pathogenic variation [26]. After symptomatic treatment and a lactose-free, rich medium-chain fatty acids dietetic treatment, the 2-year follow-up results showed that the child is growing well. HT-1 is caused by defects in enzymes in the tyrosine catabolic pathway. In our screening, only one child was ultimately diagnosed with HT-1. This child was recalled for re-screening after initial screening with both Tyr and SA greater than twice the normal value. Genetic testing was performed after the rescreening index remained elevated, and HT-1 was identified and diagnosed with a homozygous variant of the *FAH* gene c.455G > A. We immediately gave the child special formula without Tyr and Phe, mixed with breast milk daily, and oral nitisinone and hepatoprotective therapy to maintain plasma Tyr below 400 μmol/L and Phe below 120 μmol /L. We evaluated the physical and behavioural development of the children, monitored blood counts, electrolytes, and blood glucose, and followed them up until two years of age, with results indicating normal intelligence and development.

In our study, 2 cases of organic acid metabolic disorders, namely, MMA and GA-I, were diagnosed. The clinical diagnosis of MMA can be confirmed using MS/MS to detect acylcarnitines such as propinoylcarnitine (C3) and acetylcarnitine (C2), as well as amino acids such as homocysteine and methionine. Blood levels of C3 and C2 in normal neonates range from 0.30 ~ 3.00 μmol/L and 6.00 ~ 30.00 μmol/L, respectively, with C3/C2 levels ranging from 0.04 ~ 0.40 (< 0.25); methionine and homocysteine levels range from 10 ~ 35 μmol/L and 10 ~ 15 μmol/L, respectively. Blood C3 and C3/C2 are elevated in children with MMA. Blood methionine levels were decreased and homocysteine was increased in children with combined MMA. In the case of MMA, further genetic diagnosis then showed that this increase was caused by a methylmalonic acid-CoA mutase deficiency. We identified heterozygous complex variants in the *MUT* gene. c.729 730insTT (p. Asp244LeufsTer39) and c.424A > G (p. Thr142Ala). The child with MMA was mainly fed a special formula that restricted natural protein and supplemented with special formulas that removed valine, isoleucine, methionine and threonine, as well as levocarnitine (50~100 mg/kg. bid) and oral vitamin B12 (10 mg/kg. qd). MS/MS screening in children with GA-I showed elevated plasma levels of glutarylcarnitine and glutarylcarnitine /octanoylcarnitine. Cranial MRI showed significant bilateral enlargement of the temporal subarachnoid space. Based on further genetic testing and family verification, we identified complex heterozygous variants of the *GCDH* gene as c.1109 T > C (p. Leu370Pro) and c.395G > A (p. Arg132Gln), and these two mutant loci were from the father and mother, respectively. The GA-I child was mainly on a high protein restricted diet, and treatment with oral vitamin B2 (5 mg/kg. tid) with L-carnitine (50~100 mg/kg. bid). The follow-up up to the age of 2.5 years, cranial MRI showed normal and no neurological developmental deficits in these two cases.

We also diagnosed two cases of fatty acid oxidation disorder, including carnitine CPT II deficiency and PCD. CPT II deficiency is an autosomal recessive disorder caused by a lack of CPT II in the inner mitochondrial membrane, resulting in blocked transport of medium- and long-chain acyl-coenzyme A into the mitochondria for β-oxidation. In CPT II deficiency, long-chain acylcarnitine is transported across the mitochondrial membrane but is not converted to acetyl coenzyme A. Therefore, a large amount of long-chain acylcarnitine accumulates in the mitochondria. Some of the long-chain acylcarnitine is transported outside the mitochondria, resulting in a significant increase in plasma acylcarnitine, which can lead to arrhythmias, similar to those seen with carnitine transporter defects. The clinical manifestations are hypoglycaemia, coma, epilepsy, myopathy and abnormal liver function [27]. PCD is due to defects in the *SLC22A5* gene, which encodes the carnitine transporter protein, impaired renal tubular carnitine reabsorption, increased urinary carnitine loss and multiple systemic carnitine deficiencies in blood, cardiac muscle and skeletal muscle. Carnitine deficiency results in the inability of long-chain fatty acids to enter the mitochondria and participate in β-oxidation, which prevents the body from providing sufficient energy when fat mobilization is required for energy supply, and fatty acids accumulate in the cells, causing metabolic disorders and multiorgan damage. In children identified in this study, CPT II is characterized a complex heterozygous variants in the *CPT2* gene: c.989dupT (p. Ile332HisfsTer2) and c.1393G > A (p. Ala465Thr). The former is a frameshift variant, resulting in a change in synthesis from isoleucine at position 332 and an early termination codon at position 334. According to ACMG standards and guidelines, this variation is pathogenic. The latter is a missense variant, and amino acid 465 in the protein encoded by the gene transcript is changed from alanine to threonine. According to ACMG standards and guidelines, this variation is of unclear significance. One CPT II-deficient child in this study was given L-carnitine (50~100 mg/kg.bid) and formula supplemented with medium- and long-chain fatty acids to maintain stable levels of free carnitine in the blood. Follow-up results showed normal development in this child. The other case identified in this study was a PCD. In the blood tandem mass spectrometry of this child, primary carnitine C0 decreased to 2.12 μmol/L (normal reference range is 10~﻿55 μmol/L). Further genetic testing showed that SLC22A5 c.1400C > G (p. Ger467Cys) was homozygous, consistent with a pathogenic variant hotspot in China [28]. Currently, most cases of the c.1400C > G (p. Ger467Cys) variant have been reported in China [29], which may be specific to the Chinese population. The child was treated with oral L-carnitine (50~﻿100 mg/kg.bid) and milk powder with medium- and long-chain fatty acids. The child was followed up until 2 years of age, and free carnitine was maintained normally, with normal growth and mental development.

In conclusion, the retrospective analysis conducted in this study showed that our clinical treatment model of MS/MS screening combined with sequencing validation and follow-up of positive cases can effectively identify children with IEM in a timely manner and allow for the implementation of early clinical intervention, thus effectively improving the quality of life of this group of newborns. Considering the possible causes of experimental errors, the following aspects and suggestions for improvement were made. (1) The experimental cut-off values for each screening index were the same for preterm and full-term infants. Preterm infants (26.56% of total positive newborns) have different early in vivo metabolic levels than full-term infants. For example, in preterm infants, delayed maturation of the hydroxylase system may trigger hyperphenylalaninemia [30]. Therefore, the threshold for this laboratory is recommended to be determined separately for preterm infants. (2) The need for intravenous supplemental nutritional therapy, such as complex amino acids and fat emulsions, after birth due to critical factors, such as prematurity or asphyxia, can lead to abnormal neonatal disease screening results for multiple amino acids and free carnitine. The screening should be completed before supplementation with intravenous nutrition, such as complex amino acids and fat emulsions, or screening should be promptly reviewed after stopping intravenous nutrition treatment if conditions permit. In total, 175 cases had increased free carnitine levels, accounting for 12.30% of cases, which is higher than the national average figure. Considering the association with the early use of levocarnitine (carnitine) in preterm infants, a review after stopping the drug showed that free carnitine decreased to normal levels in most infants. (3) Some newborns did not eat well, and their families did not breastfeed them adequately during the prescribed time. Due to insufficient protein intake, the blood was low in various amino acids and other metabolites, resulting in abnormal screening results. According to the relevant requirements of the Technical Specification for Blood Tablet Collection for Newborn Screening [31], blood should be collected after adequate breastfeeding.

## Data Availability

The datasets used and/or analysed during the current study are available from the corresponding author on reasonable request.

## Abbreviations

IEM: Inborn errors of metabolism
MS/MS: Tandem mass spectrometry
NBS: Newborn screening
DBS: Dried blood spots
EQA: External quality assessment
PKU: Phenylketonuria
HT-1: Tyrosinemia type I
PAH: Phenylalanine hydroxylase
Phe: Phenylalanine
PCD: Primary carnitine deficiency
Tyr: Tyrosine
NICCD: Neonatal intrahepatic cholestasis caused by citrin deficiency
MCT: Middle-chain triglyceride
HT: Hereditary tyrosinemia
FAH: Fumarylacetoacetate hydrolase
SA: Succinylacetone
MMA: Methylmalonic acidemia
GA-I: Glutaric aciduria type I
CPT II: Carnitine palmitoyltransferase II
